# Impending Death from the Inhalation of Chloroform

**Published:** 1853-11

**Authors:** 


*Impending Death from the Inhalation of Chloroform; Recovery by the direct Insufflation from Mouth to Mouth, according to Mr. Ricords Method.*

M. Boinet has just published in the *Bulleiin de Therapeutique* the case of a lady, 30 years of age, who inhaled choloroform to escape the pain of having the forceps applied. She was short and ill-proportioned, and it had been a question whether the natural term of gestation should be waited for, or whether premature labor should be induced. The former course was adopted, put the head became locked at the outlet of the pelvis. Before the forceps were used, a cambric handkerchief, upon which about two drachms of choloroform had been poured, was held at a short distance from the patients nose. She was soon quite insensible, without experiencing any excitement; and as the operation was being prolonged, M. Boinet desired the husband again to place the handkerchief to the patients mouth, as she was beginning to move about, and to utter faint cries. The transaction was now renewed, and the child extracted; but the husband being engrossed by the operation and the perilous situation of his wife, left the handkerchief upon her face, though he had been recommended to take it off immediately the patient was again insensible. Just as the child was being withdrawn, the assistant who was watching the pulse, stated that it had stopped. The cord was quickly cut, the child removed, and the windows thrown wide open. The heart had ceased to beat, the most complete relaxation of the frame existed, the pallor was extreme; in fact, all the characteristics of death had made their appearance. For five minutes cold water, ammonia, slapping &c., were used with the greatest solicitude, but in vain, and the patient was declared dead. M. Boinet thought, however, that he would not give up the case until he had tried the mouth to mouth insufflation. This mode of filling the lungs with air remained at first ineffectual, as the cheeks flapped back immediately the blowing ceased; bellows were also tried, but to no better purpose And now, finally, as M. Boinet states, a few more direct insufflations were attempted; more to avoid the imputation of leaving the patient too soon than from any hope of recovering her. These were then continued with great energy, while the assistant pressed the lower part of the thorax, to excite the diphragm to action, At last the patient made an inspiration, which the author compares to the last gasp of a dying person; and he continued the insufflations without much hope of observing a second inspiration, as the pulse and heart were quite still. But in a few seconds a second breathing effort took place, and the patient gradually recovered, exactly as happens with those who wake from their narcotic sleep without having experienced any arrest of circulation. When the patient was quite recovered, the placenta was taken away, and she made a very good recovery. The question now arises whether this was a mere swoon, which would have been recovered from without any efforts at insufflation.

